# Publication ethics standards should be the same for all peer-reviewed journals

**DOI:** 10.3325/cmj.2017.58.195

**Published:** 2017-04

**Authors:** Armen Yuri Gasparyan

The letter-to-the-editor by S.Grazio and B.Anic highlights the importance of ethical editorial policies for non-English journals. There are still numerous local and regional journals which opt to publish their articles in local languages with English abstracts. Readers of such journals are in dire need of articles presenting new knowledge and progress in science, education, and practice in an understandable language. Visibility of such journals is not high, but it can be improved by adhering to the international standards of research reporting, referencing, disclosing ethical issues, and properly digitizing metadata and author IDs. The Sarajevo Declaration on Integrity and Visibility of Scholarly Publications (DIVA) was drafted by editors from Balkan and Mediterranean countries to increase awareness of authors, reviewers, and editors of acceptable publication practices in the region (1). Although updated recommendations of global editorial associations are widely available and there is no shortage of institutional research and publication policies, some regional publishers continue ignoring them and publishing useless, redundant, and apparently unethical original research and reviews. Unaware editors of Scopus and Web of Science-indexed journals continue accepting articles supplied by commercial brokering agencies that violate norms of disclosing authorship and conflicts of interest. Some agencies even promise to publish their clients' works by helping them to somehow survive the peer review.

The statements of the Declaration are supposed to address the issues of both English and non-English non-mainstream science journals because publication standards should be the same for all types of peer-reviewed journals. Non-English journals are overlooked by most leading publication experts simply because they do not read these sources of information. But the problem is that prestigious multidisciplinary and specialist databases, such as Scopus and MEDLINE, index both English and non-English sources and unethical authors and agencies can indiscriminately target both types of journals. It is expected that the initiative of the authors of the Declaration will be supported by all regional editors who aim to upgrade their instructions for authors and publish properly checked and edited papers. The Declaration is also a reminder to researchers and academicians from non-mainstream science countries that they need to improve their proficiency in research reporting in any language and target ethical journals. They should be also aware of the blacklisted indexed journals and publishers. Although Jeffrey Beall’s list vanished from his Scholarly Open Access blog in January 2017, it is still available from back-up sites (2).
